# Multiplex PCR-based titration (MPBT) assay for determination of infectious titers of the three Sabin strains of live poliovirus vaccine

**DOI:** 10.1186/s12985-019-1233-6

**Published:** 2019-10-28

**Authors:** Hasmik Manukyan, Elvira Rodionova, Tatiana Zagorodnyaya, Tsai-Lien Lin, Konstantin Chumakov, Majid Laassri

**Affiliations:** Center for Biologics Evaluation and Research, US Food and Drug Administration, 10903 New Hampshire Avenue, Silver Spring, MD 20993 USA

**Keywords:** Multiplex titration, Poliovirus surveillance, Virus excretion, Clinical trials, Mucosal immunity

## Abstract

**Background:**

Conventional assays to titrate polioviruses usually test serial dilutions inoculated into replicate cell cultures to determine a 50% cytopathic endpoint, a process that is both time-consuming and laborious. Such a method is still used to measure potency of live Oral Poliovirus Vaccine during vaccine development and production and in some clinical trials. However, the conventional method is not suited to identify and titrate virus in the large numbers of fecal samples generated during clinical trials. Determining titers of each of the three Sabin strains co-existing in Oral Poliovirus Vaccine presents an additional challenge.

**Results:**

A new assay using quantitative multiplex polymerase chain reaction as an endpoint instead of cytopathic effect was developed to overcome these limitations. In the multiplex polymerase chain reaction-based titration assay, cell cultures were infected with serial dilutions of test samples, lysed after two-day incubation, and subjected to a quantitative multiplex one-step reverse-transcriptase polymerase chain reaction. All three serotypes of poliovirus were identified in single samples and titers calculated. The multiplex polymerase chain reaction-based titration assay was reproducible, robust and sensitive. Its lower limits of titration for three Sabin strains were 1–5 cell culture 50% infectious doses per ml. We prepared different combinations of three Sabin strains and compared titers obtained with conventional and multiplex polymerase chain reaction-based titration assays. Results of the two assays correlated well and showed similar results and sensitivity. Multiplex polymerase chain reaction-based titration assay was completed in two to 3 days instead of 10 days for the conventional assay.

**Conclusions:**

The multiplex polymerase chain reaction-based titration (MPBT) is the first quantitative assay that identifies and titrates each of several different infectious viruses simultaneously in a mixture. It is suitable to identify and titrate polioviruses rapidly during the vaccine manufacturing process as a quality control test, in large clinical trials of vaccines, and for environmental surveillance of polioviruses. The MPBT assay can be automated for high-throughput implementation and applied for other viruses including those with no cytopathic effect.

## Background

Poliomyelitis is a highly contagious neurological disease caused by three distinct serotypes of poliovirus. There are two excellent vaccines that protect against poliomyelitis: inactivated poliovirus vaccine (IPV) and live oral poliovirus vaccine (OPV). Both vaccines played key roles in eliminating paralytic poliomyelitis from most countries worldwide.

The World Health Organization’s (WHO) Global Polio Eradication Initiative (GPEI) has made significant progress toward eradicating the disease. It has stopped transmission of wild polioviruses types 2 and 3 [1] and eliminated wild type-1 poliovirus except for endemic regions in Afghanistan and Pakistan. This remarkable progress was achieved using multiple doses of trivalent live attenuated oral poliovirus vaccine (OPV), which stimulates robust systemic and mucosal immunity [2] and serves to immunize close contacts as well as vaccinees themselves. All Recipients of OPV excrete mutant variants of Sabin strains in stools; those vaccine-derived polioviruses can evolve into pathogenic variants that circulate in the population (cVDPV) and infect unvaccinated people [3]. Unfortunately, about one in every million OPV vaccinees and their contacts have developed vaccine-associated paralytic poliomyelitis (VAPP), an unacceptable complication in countries from which the wild-type viruses have been eradicated. In addition, persons with certain kinds of B-cell immunodeficiency can become chronically infected and excrete vaccine-derived virus for a long time [4–7]. Consequently, many countries stopped vaccinating with OPV. To address this issue, in April 2016 the WHO recommended gradually phasing out use of trivalent OPV and replacing it with a bivalent OPV (bOPV) containing only serotypes 1 and 3, plus administering at least one dose of IPV [8, 9]. An important limitation of IPV is that it elicits poor intestinal immunity. Therefore, children receiving bOPV with only limited number doses of IPV may not acquire sufficient mucosal immunity to prevent infections with type-2 poliovirus, putting them at risk for paralytic disease and amplifying transmission of the type 2 of poliovirus, should it be reintroduced into the environment [10–12]. Efforts are now underway to improve IPV’s ability to induce mucosal immunity [13] but those preparations of IPV are not yet available. Better methods to evaluate mucosal immunity will be increasingly important to evaluate improved vaccines. A straightforward way to evaluate intestinal immunity might be to challenge IPV recipients with OPV and then identify and titrate the viruses they excrete in stool. Surveillance of polioviruses in patients with acute flaccid paralysis and in sewage samples are also important parts of any polio eradication program. In addition, industry needs better tests to identify, titrate and estimate dynamic inactivation of polioviruses during vaccine production. Any new test for polioviruses should be rapid, robust, have high throughput, and offer multiplex simultaneous detection and accurate titration for each of the three serotypes of poliovirus.

Currently many viruses are quantified by traditional culture in susceptible cell monolayers, either by plaque assay or by quantal terminal-dilution in multi-well plates. Plaque assays require tedious visual counting of plaques that vary in size, complicating automated reading. Terminal-dilution quantal methods require monitoring of cytopathic effects (CPE), often over a long period [14–16]. In addition, when several virus types are present in a mixture, all other viruses except the one of interest must be neutralized, further complicating the task. Therefore, conventional virus titration techniques are not well suited to screen the many samples generated during clinical vaccine trials, environmental surveys, and quality control of vaccine production [17].

We previously compared a real-time PCR-based assay (osRT-PCR) with a conventional quantal terminal-dilution method using CPE-based endpoint assay (CCID_50_). Cells were infected with poliovirus followed by PCR quantitation of viral RNA in cell lysates before CPE appeared [18]; both methods yielded similar results. Based on that observation, we developed a multiplex PCR-based titration (MPBT) assay that facilitates rapid titrations of OPV strains. Hep-2C cells in 96-well plates are exposed to serial dilutions of virus followed by 2 days of incubation. Cell lysates are then subjected to multiplex quantification by qmosRT-PCR [19]. Endpoints obtained using this PCR-based assay were compared to those from conventional CCID_50_ assays and found to be similar. The MPBT method is simple, rapid, robust, reproducible, sensitive, and suitable for multiplex titration of viruses.

## Results

### Comparative titrations of different lots of Sabin OPV strains with MPBT and CCID_50_ assays

Four different lots of Sabin strains were titrated in simple format (only one virus titrated in each run) and in multiplex format (all three Sabin OPV strains were mixed and titrated simultaneously as one sample) using both MPBT and CCID_50_ assays for simple format and MPBT assay alone for the multiplex format. Results are summarized in Table 1. The titers calculated for each virus were similar for both assays and for both monotype and multiplex formats. The four lots of Sabin OPV strains analyzed in this experiment had high titers, around 8 log_10_ CCID_50_/ml, and similar titers were obtained by MPBT and CCID_50_ assay for samples with titers less than 100 CCID_50_ /ml (see Table 2). These results show that the MPBT assay generated results similar to those of the CCID_50_ assay and worked well for multiplex titration of OPV viruses.

**Table 1:** Titration of different lots of Sabin strains with both MPBT and CCID50 assays

Sabin type (lot)	Single titration^a^		Multiplex titration^a^	Mean	SD	RSD (%)
	MPBT assay	CCID50 assay	MPBT assay			
1 (1)	8.75	8.75	9.13	8.88	0.22	53.92
2 (1)	8.63	8.63	8.63	8.63	0.00	0.00
3 (1)	9.36	9.25	9	9.20	0.18	44.47
1 (2)	8.6	8.63	8.62	8.62	0.02	3.52
2 (2)	8.19	8.04	8.18	8.14	0.08	19.49
3 (2)	8.57	8.42	8.49	8.49	0.08	17.41
1 (3)	8.52	8.43	8.56	8.50	0.07	15.42
2 (3)	8.31	7.97	8.26	8.18	0.18	44.23
3 (3)	8.43	8.39	8.34	8.39	0.05	10.41
1 (4)	7.77	7.77	8.07	7.87	0.17	41.52
2 (4)	7.73	7.65	8.14	7.84	0.26	66.52
3 (4)	8.1	7.94	7.84	7.96	0.13	30.90

^a^The titers are in Log10CCID50/ml, *RSD* Relative standard deviation, *SD* Standard deviation of log10 titers, Single titration; Each Sabin Strain was titrated separately, Multiplex titration; All 3 Sabin strains were titrated in the same reaction

**Table 2:** Determination of the low limit of titration (LOT) of OPV viruses by MPBT assay and its comparison with LOT of CCID50 assay

Assay	Sabin strain combinations	Titer of the analyzed viruses (CCID_50_/ml)						
		100	50	25	10	5	1	0.1
MPBT	1	125.89	81.28	35.48	31.62	NT	19.95	UD
CCID50		158.49	87.10	50.12	22.39	15.85	17.78	UD
MPBT	2	123.03	51.29	28.18	22.39	17.78	UD	UD
CCID50		95.50	36.31	35.48	19.95	17.78	UD	UD
MPBT	3	104.70	56.23	30.90	15.85	19.95	UD	UD
CCID50		95.50	51.30	33.88	16.98	15.85	UD	UD
MPBT	1 2 3	104.71	39.81	36.31	17.78	15.85	UD	UD
		74.13	74.13	21.88	19.95	17.78	UD	UD
		56.23	28.18	21.88	19.95	15.85	UD	UD

*NT* Not tested, *UD* Undetermined

### MPBT specificity, sensitivity, and ability to determine amounts of each Sabin OPV strain in a mixture

Previously we characterized qmosRT-PCR and generated standard calibration curves by testing Sabin viruses of known titers (expressed as CCID_50_/ml) [19]. RNA was extracted from the three OPV viruses and serial ten-fold dilutions prepared from individual virus RNAs, combined RNA from two viruses, and combined RNA from all three viruses; samples were subjected to quantitative simplex one-step RT-PCR, duplex one-step RT-PCR, or triplex one-step RT-PCR, depending on the combinations of RNAs tested in the same reaction to generate standard curves. All curves showed good linearity with R-squared values exceeding 0.95. The linear ranges were 9 log_10_ for single-type PCR, 8–9 log_10_ for duplex PCR and 7–8 log_10_ for triplex PCR. These results showed that both monospecific and multiplex PCRs were very specific and sensitive. The limit of quantification (Based on viral RNA quantification) of qmosRT-PCR for three Sabin OPV strains mixed together fell between 0.29–2.86, 0.13–1.26 and 0.36–3.60 CCID_50_/ml for types 1, 2, and 3 respectively [19].

In this work, virus dilutions containing 0.1 to 100 CCID_50_/ml were used to determine the sensitivity of the MPBT assay. For single-virus titrations, we compared MPBT and CCID_50_ assays. Results, summarized in Table 2, showed that the limit of titration (LOT) of single-virus titrations were 0.1 to 1 CCID_50_/ml for Sabin 1 and 1 to 5 CCID_50_/ml for Sabin 2 and 3 for both MPBT and conventional CCID_50_ assays. When all three Sabin strains were titrated together in the same reaction, LOTs of the MPBT assay were 1–5 CCID_50_/ml for Sabin 1, 2, and 3. Both assays had similar sensitivity for titrations of a single virus. While CCID_50_ assays cannot titrate more than one virus per reaction, the MPBT assays were able to titrate each Sabin strain mixed in the same sample with high sensitivity and specificity.

### Correlations between MPBT and CCID_50_ assays

**Fig. 1:**
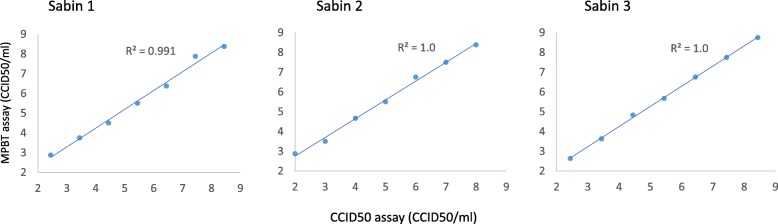
Evaluation of correlations between MPBT and CCID_50_ assays using three Sabin strains with known titers (determined by CCID_50_ assay)

We assessed correlations between MPBT and CCID_50_ assays using three samples (one for each Sabin strain) with titers previously determined by CCID_50_ assay. The viruses with known titers were mixed, serially diluted ten-fold, and each dilution subjected to MPBT assay titration as described above. The results of MPBT assays plotted against the known CCID_50_ titers of the corresponding dilutions for each Sabin strain are presented in Fig. 1. Excellent correlations were observed between the two assays for each of the three Sabin strains with R-squared value of 0.99 for Sabin 1 virus and 1.0 for Sabin 2 and 3 viruses. The estimated slopes and associated 95% confidence intervals are 0.95 (0.85, 1.06), 0.95 (0.88, 1.02), and 1.02 (0.99, 1.05) for Sabin 1, 2, and 3 viruses, respectively, suggesting no proportional bias between the two methods.

### Consistency of MPBT assay

To assess consistency of the MPBT assay, ten-fold dilutions, we titrated one lot of Sabin 1, 2 and 3 mixture simultaneously five times on the same day. On another day, the same operator titrated the same lot of the three Sabin strains simultaneously five times using two-fold dilutions. Result of these experiments, summarized in Table 3, showed that the MPBT assay generated consistent results for all three Sabin strains: the repeats with ten-fold dilutions and with two-fold dilutions. We observed no significant differences in titers repeatedly obtained with ten-fold and two-fold dilutions. The maximum percent of relative standard of variation observed was 65 for Sabin 2 virus repeatedly titrated with ten-fold dilutions. in short, the MBPT assay yielded consistent and reproducible titers.

**Table 3:** Results of consistency evaluation of MPBT assay (titers are expressed on log10CCID50/ml)

Sabin strain	Repeat codes					Mean	SD	RSD %
	1	2	3	4	5			
Sabin 1^a^	8.75	8.63	9.10	8.75	8.75	8.80	0.18	42.69
Sabin 1^b^	8.66	8.77	8.84	9.07	8.51	8.77	0.21	51.03
Sabin 2^a^	8.63	8.63	8.75	9.25	8.75	8.80	0.26	64.91
Sabin 2^b^	8.28	8.39	8.51	8.51	8.28	8.39	0.12	26.96
Sabin 3^a^	8.88	8.75	8.88	9.00	9.25	8.95	0.19	45.56
Sabin ^b^	8.32	8.13	8.43	8.24	8.35	8.29	0.11	26.74

*SD* Standard deviation of log10 titers, *RSD* Relative standard deviation

^a^Virus was titrated with 10-fold dilution

^b^virus was titrated with 2-fold dilution

### Robustness of virus titrations by the MPBT assay

We studied the robustness of the MPBT assay to ensure that measurements of virus titer remained unaffected by small variations deliberately introduced into the procedure. To do this, we performed MPBT assays for the three Sabin strains simultaneously but seeded different numbers of cells into the wells (1–4 × 10^4^). Results of these titrations, summarized in Table 4, showed that, despite four-fold variation in cell numbers per well, MPBT assays generated similar results, with relative standard deviation percentages for each virus of 88 for Sabin 1, 16 for Sabin 2 and 36 for Sabin 3.

**Table 4:** Evaluation of the effect of cell numbers per well variation on the MPBT assay results (log10CCID50/ml)

Sabin Strain	Cell numbers per well			Mean	SD	RSD %
	1 10^4^	2 10^4^	4 10^4^			
1	8.5	9.12	8.62	8.75	0.33	87.98
2	8.5	8.38	8.5	8.46	0.07	16.05
3	8.62	8.88	8.88	8.79	0.15	35.62

*SD* Standard deviation of log10 titers, *RSD* Relative standard deviation

We also investigated effects of delay between diluting virus and adding cells in MPBT assays. Delays of 15 min to 1 h showed no effect on results. Titers, summarized in Table 5, were similar after delays of 15, 30, 45 and 60 min, demonstrating that the MPBT assay was robust when all three Sabin strains were titrated simultaneously in the same reaction.

**Table 5:** Evaluation of the effect of delay between diluting virus and adding cells on MPBT assay results (log10CCID50/ml)

Sabin Strain	Delay in minutes				Mean	SD	RSD %
	15	30	45	60			
1	8.75	8.88	9.13	8.88	8.91	0.16	37.86
2	8.75	8.63	8.63	8.50	8.63	0.10	23.84
3	9.25	8.88	9.13	9.00	9.07	0.16	38.15

*SD* Standard deviation of log10 titers, *RSD* Relative standard deviation

### MPBT assays with blind samples

To validate MPBT assay, polioviruses of all three serotypes with known CCID_50_ titers were mixed in different combinations and concentrations and tested by a single operator using the MPBT assay in blind format. The results are presented in Table 6. The assay specifically identified each type of Sabin OPV virus in all combinations, and the titers for each Sabin strain were those expected based on the dilution tested.

**Table 6:** Virus titer determination in samples prepared with different combination of Sabin strains and analyzed in blind format with MPBT assay

Samples codes	Spiked CCID50 titer^a^ (Sabin type)	Results of MPBT assay	
		Sabin type	Titer^a^
A	7.63 (1)	1	7.5
B	7.04 (2)	2	7
C	7.42 (3)	3	7.75
D	7.63 (1)	1	7.38
	7.04 (2)	2	6.75
E	7.63 (1)	1	7.38
	7.42 (3)	3	7.5
F	7.04 (2)	2	6.88
	7.42 (3)	3	7.25
G	7.63 (1)	1	7.25
	7.04 (2)	2	7.25
	7.42 (3)	3	7.75

^a^Titers of Sabin strains are expressed on log10CCID50/ml

### MPBT assays of stool samples collected from a clinical trial of OPV2

To determine the ability of the MPBT assay to titrate OPV virus strains in clinical samples, we used supernatants of stool samples collected during a clinical trial of monovalent OPV2, part of the FIDEC study [20]. Samples were diluted two-fold with DMEM and filtered through 0.22-μm spin-tube filters (COSTAR). The filtrates were analyzed with both MPBT and CCID_50_ assays. Results, summarized in Table 7, show that titers for each sample were similar with both assays. This finding demonstrates that the MPBT assay can be used to detect and titrate OPV virus shed in the stool during clinical trials and in the environment.

**Table 7:** Analysis of stool samples collected from clinical trial of OPV2 with MPBT and CCID50 assays

Stool sample codes	MPBT assay (log_10_CCID_50_/ml)	CCID50 assay (log_10_CCID_50_/ml)
1	3.43	3.51
2	3.77	3.29
3	3.86	3.67
4	4.05	3.9
5	2.1	2.23
6	2.49	2.81
7	3.55	3.43

## Discussion

Sensitive, specific, reproducible and high-throughput assays of infectious viruses are needed to detect polioviruses in clinical and environmental samples and essential to monitor the manufacturing of live and inactivated poliovirus vaccines [21]. The viral plaque assay was developed in 1952 by Renato Dulbecco and became widely used to quantify viruses [22–25]. It is one of the most widely used method in virology to purify a clonal population of virus or to determine viral titer as plaque formation unite (PFU) per milliliter [26–30]. Since most plaques contain the progeny of a single virus particle, clonal populations can be purified by isolating virus from individual plaques. However, it is usually necessary to stain monolayers to enhance the contrast between adjacent living cells and the plaques. Therefore, viral plaque assays are typically time-consuming, generally requiring four to 10 days, depending on the virus. Furthermore, plaque assays work only for viruses capable of infecting cells in adherent monolayers, multiplying and lysing them; some viruses do not form plaques in culture. CCID_50_ assays are also used to titrate viruses that cause CPE in tissue cultures—usually over five to 20 days—and may be effective even when many cells in the culture remain viable. CCID_50_ titrations are also relatively cheap, economical of experimental materials and reagents and other consumable supplies, and easy to analyze [27]. However, not all viruses cause CPE in cells they infect. Results of both plaque and quantal CCID_50_ assays are somewhat variable; relative errors with the plaque assay can be more than 10%, and CCID_50_ titers often have 35% error rate [31]. In general, CCID_50_ assays are used more often than plaque assays to titrate viruses for research and diagnosis [27, 31–33], but both are difficult to automate, and neither is especially suitable for large-scale analyses of clinical and environmental samples. Without further modification, neither assay deals easily with samples containing more than one virus, such as trivalent OPV viruses.

Quality control of poliovirus vaccine production, including in-process confirmation of virus identity and study of viral inactivation dynamics, also requires rapid testing of many samples. Manufacturers use seroneutralization identity testing of two serially diluted samples and a reference virus in cell culture medium tested with and without addition of specific monoclonal antibodies. The identity of the serotype is determined from the difference between infectivity titers of samples with and without specific antibodies [34, 35]. This method is time-consuming, labor-intensive, and error-prone, sometimes yielding false negative results. To overcome these limitations, encouraged by our previous finding that real-time PCR quantitation of virus from lysates of infected cell cultures gave results similar to those with a conventional CCID_50_ assay [18], we developed a quantitative multiplex one-step RT-PCR (qmosRT-PCR) [19] and a multiplex PCR-based titration (MPBT) assay for multiplex simultaneous titration of all three infectious Sabin OPV poliovirus strains.

Similar to CCID_50_ assays, the MPBT assay tests serially diluted samples of virus mixed with susceptible cells in replicate wells of 96-well plates incubated at 36 °C, except that instead of waiting for the appearance of CPE, medium is discarded after 36–42 h of incubation, cells lysed with 0.9% triton, and then viral nucleic acids in cell lysates are quantified by qmosRT-PCR assay; results are used to determine virus titers expressed as CCID_50_ units. Therefore, virus titer is not determined based on proportionality to Ct values obtained by qmosRT-PCR, but rather qmosRT-PCR is used as a readout method to determine the presence or absence of viral replication. Therefore qmosRT-PCR results are expressed in yes / no format, allowing a simple Karber formula to be used similar to conventional CCID_50_ assay. As a result this assay detects only live viruses and not nucleic acids that may be present in dead particles.

MPBT proved to be very sensitive, detecting an equivalent of 1–5 CCID_50_/ml of OPV types 2 and 3 alone and OPV types 1, 2 and 3 in mixtures and 0.1–1 CCID_50_/ml of OPV type 1 virus tested alone. The MPBA assay detected OPV types 1, 2 and 3 alone with sensitivities similar to those of the CCID_50_ assay (Table 1). In addition, the MPBT method generated titers similar to those of CCID_50_ assays for all combinations of Sabin OPV poliovirus strains s (Tables 2 and 6).

We found excellent correlations between the two assays for all the three Sabin OPV poliovirus strains with an R-squared value of 0.99 for Sabin 1 and R-squared values of 1.0 for each of Sabin 2 and 3 OPV polioviruses (Fig. 1). The MPBT assay generated consistent results for all three Sabin OPV poliovirus strains (Table 3). The MPBT assay proved to be very robust; even a one-hour delay between diluting the viruses and adding cells and a four-fold variation in numbers of cells added had no effect on titers of viruses (Tables 4 and 5). This method reduced the time needed to titrate OPV polioviruses from the seven to 10 days for conventional CCID_50_ to only two to 3 days for MPBT. In contrast to CCID_50_ assay, the MPBT assay accurately titrated all three Sabin OPV poliovirus strains mixed in the same sample.

## Conclusions

The MPBT assay described in this communication offers a simple and rapid alternative to traditional CCID_50_ assays to detect, identify, and titrate either individual or combined serotypes of Sabin OPV polioviruses. The MPBT method is well suited to detect and quantify polioviruses in the many fecal samples collected during clinical trials of new poliovirus vaccines (IPV/OPV), and during routine clinical and environmental poliovirus surveillance programs. The MPBT assay can also be applied during manufacture of poliovirus vaccines, for in-process quality control by identifying serotypes of vaccine polioviruses (potentially replacing the current seroneutralization identity test) and monitoring inactivation dynamics. This assay is suitable to automate, facilitating high-throughput applications, improving consistency of titrations, and saving time, cost, and labor. The MPBT method can also be applied to detect and titrate other viruses, including those that produce no CPE.

## Methods

### Viruses, Hep-2C cells and clinical samples

Lots of US neurovirulence poliovirus reference vaccines (containing Sabin type 1, 2, and 3 OPV strains having GenBank accession numbers: AY184219, AY184220, and AY184221 respectively) were used to compare results of CCID_50_ and MPBT assays.

HEp-2C cells (ATCC® CCL-23™), derived from a human carcinoma, were cultured in 175-cm^2^ flasks at + 37 °C ± 2 °C in Dulbecco’s modified eagle medium (DMEM; Gibco) supplemented with 5% fetal calf serum (FCS; Gibco) and penicillin-streptomycin (100 U/mL and 100 μg/ mL, respectively; Gibco). Viable cells were sampled, stained with Trypan Blue (Invitrogen) and counted with a hemocytometer (Countess™; ThermoFischer). Hep-2C cells were used for both the CCID_50_ and MPBT assays.

Anonymized stool samples used in this work were collected from a clinical trial of OPV2 (“Fighting Infectious Diseases in Emerging Countries” [FIDEC]) [20] that was approved by ethics committees and as appropriate by National Regulatory Authorities, the Colorado Multiple Institutional Review Board, and the Western Institutional Review Board.

### Virus titration by CCID_50_ assay

Virus strains used in OPV were quantified by endpoint dilution titration on HEp-2C cells and the results expressed as log_10_ CCID_50_/mL. (CCID_50_ is cell culture 50% infectious dose, defined as that dilution of virus required to infect 50% of the cell monolayers.) Serial ten-fold or two-fold dilutions of viral samples were prepared in 100 μl of DMEM supplemented with 2% FCS in 96-well plates. One-hundred-μl aliquots of cell suspension containing 2 × 10^4^ HEp-2C cells in DMEM with 2% FCS were added to each well of diluted virus in replicate wells of 96-well plates. Virus-infected plates were incubated for 10 days at 36 °C in a 5% CO_2_ humid atmosphere; wells were periodically inspected for CPE. Wells showing CPE were counted on day ten after infection and virus titers calculated using the Spearman-Karber formula [36].

### Multiplex PCR-based titration (MPBT) assay

Viruses were diluted and mixed with cells in 96-well plates as for the CCID_50_ assay described above, with the following exceptions: plates were incubated for only 42 h, after which the medium was discarded and 50-μl aliquots of lysis solution (DMEM with 0.9% Triton X-100) were added to each well. The plates were then sealed with foil and stored at -80 °C prior to quantitative multiplex one-step RT-PCR (qmosRT-PCR) analysis [19].

The plates with the triton-lysed cells were thawed for 30 min at room temperature and briefly centrifuged in a 5810R centrifuge (Eppendorf, San Diego, CA) at 1000 rpm for 1 min to collect the lysate at the bottom of the wells. Dilutions of 1:10 were then prepared by adding 90 μl of molecular biology grade water (5-PRIME, Gaithersburg, MD) to 10 μl of the cell lysates.

All three poliovirus serotypes were quantified in the same qmosRT-PCR reaction as described previously [19]. Briefly, TaqMan probes with different dyes (FAM, VIC, and NED fluorescent reporters and a non-fluorescent quencher; Applied Biosystems) were used in the qmosRT-PCR to discriminate between different poliovirus serotypes. Two microliters of diluted lysate were added to each well of a PCR plate, and the multiplex quantitative RT-PCR assay was performed as described [19]. The samples that have Cts less or equal than 40 are considered positive and samples with Cts higher than 40 are considered negative. The PCR reaction considered non-valid if positive control was negative and/or negative control was positive. Each well of the plate was scored as positive or negative for the presence of the virus activity and used for titers calculation of each OPV serotype according to Spearman-Karber formula [36]. The Spearman-Karber formula used is as follows: log CCID50 = −(L-d(S-0.5)) where, L is the log of lowest dilution in test, d is the difference between log dilutions and S is the sum of proportions of positive tests (i.e. cell cultures positive for the presence of the virus by PCR).

## Data Availability

The datasets generated during and/or analyzed during the current study are available from the corresponding author on reasonable request.

## Abbreviations

bOPV: Bivalent oral poliovirus vaccine
CCID50: Cell culture infectious dose 50%
CPE: Cytopathic effect
GPEI: Global Polio Eradication Initiative
IPV: Inactivated poliovirus vaccine
MPBT: Multiplex PCR-based titration
OPV: Trivalent oral poliovirus vaccine
PFU: Plaque formation unite
qmosRT-PCR: Quantitative multiplex one-step reverse-transcriptase polymerase chain reaction
RSD: Relative standard deviation
RT-PCR: Reverse-transcriptase polymerase chain reaction
WHO: World Health Organization
